# Therapeutic Use of Cannabidiol in Autism Spectrum Disorder in the Pediatric Population. Literature Review

**DOI:** 10.1192/j.eurpsy.2026.10554

**Published:** 2026-07-31

**Authors:** I. M. Peso Navarro, C. Garcia Cerdan, M. Ligero Argudo, I. Rodriguez Blanco, J. I. de la Iglesia Larrad, R. K. Gonzalez Bolaños

**Affiliations:** Psiquiatria, Complejo Asistencial Salamanca, Salamanca, Spain

## Abstract

**Introduction:**

Autism spectrum disorders (ASD) are neurodevelopmental conditions marked by social communication difficulties and repetitive behaviors, affecting 1–2% of children and adolescents.

The endocannabinoid system (ECS), important for brain function, is altered in individuals with ASD. Cannabidiol (CBD), a non-psychoactive compound from cannabis, modulates the ECS and other systems, with anti-inflammatory, anxiolytic, and neuroprotective effects.

Though evidence is still limited, some studies suggest CBD may help improve behavior, anxiety, sleep, and hyperactivity in children with ASD, with generally mild side effects. CBD is emerging as a promising treatment option, but more research is needed.

**Objectives:**

**General Objective**

The main objective of this literature review is to gather and analyze the published evidence regarding the potential therapeutic uses of CBD in ASD in the child and adolescent population.

**Specific Objectives**

1. To investigate the clinical efficacy of CBD in ASD in the child and adolescent population.

2. To explore the side effects and safety associated with cannabidiol treatment.

**Methods:**

A literature review was conducted following PRISMA guidelines, focusing on studies involving children and adolescents (0–18 years) with ASD treated with CBD, with or without low doses of THC. The search was carried out in **PubMed** and **Scopus** between **October 2024 and February 2025**, using Boolean operators and terms related to CBD, ASD, and the pediatric population. After filtering and removing duplicates, **12 articles** were selected for review.

**Results:**

The therapeutic use of CBD for ASD in children and adolescents is a recent and emerging area. This review included 12 studies published between 2019 and 2025, mostly observational in nature, with only one randomized controlled trial.

A total of 1,026 participants, aged 2 to 18 years, were studied. Most studies used CBD combined with low doses of THC, and only one used purified CBD. Treatment was given alongside existing medications and sometimes allowed dose reductions.

Follow-up periods ranged from 3 to 28 months. Side effects were generally mild to moderate, including restlessness, irritability, and changes in appetite or sleep. Overall, CBD showed good safety and tolerability.

**Conclusions:**

1. **CBD shows potential** in improving symptoms of ASD, especially in enhancing social interaction.
2. It may be a **useful option for treatment-resistant cases** or severe behavioral issues.
3. Studies suggest benefits in managing **common comorbidities** like anxiety, hyperactivity, and sleep disturbances.
4. **Safety profile is generally good**, with mostly mild side effects, but close monitoring is necessary.
5. Current evidence is **limited and heterogeneous**; more rigorous, large-scale studies are needed.
6. Future research should **standardize protocols**, explore long-term effects, and clarify dosing and drug interactions.

**Disclosure of Interest:**

None Declared

